# A guide to selecting high-performing antibodies for STING1 (Uniprot ID: Q86WV6) for use in western blot, immunoprecipitation, and immunofluorescence

**DOI:** 10.12688/f1000research.155929.1

**Published:** 2024-09-12

**Authors:** Vera Ruíz Moleón, Charles Alende, Maryam Fotouhi, Riham Ayoubi, Kathleen Southern, Carl Laflamme, NeuroSGC/YCharOS/EDDU collaborative group, ABIF consortium

**Affiliations:** 1 Department of Neurology and Neurosurgery, Structural Genomics Consortium, The Montreal Neurological Institute, McGill University, Montreal, Québec, H3A 2B4, Canada

**Keywords:** Uniprot ID: Q86WV6, STING1, hSTING, MPYS, MITA, stimulator of interferon genes protein, mediator of IRF3 activation, transmembrane protein 173, antibody characterization, antibody validation, western blot, immunoprecipitation, immunofluorescence

## Abstract

STING1 is an immune adaptor protein which promotes innate immune defense mechanisms against pathogens. Its role in modulating inflammation links STING1 to various pathologic conditions, positioning it as a key target for therapeutic interventions aimed at regulating immune responses. To advance our understanding of STING1-associated diseases, it is essential to make high-performing antibodies readily accessible to the scientific community. This study aims to improve reliability of STING1 research as we have characterized sixteen STING1 commercial antibodies for western blot, immunoprecipitation, and immunofluorescence using a standardized experimental protocol based on comparing read-outs in knockout cell lines and isogenic parental controls. These studies are part of a larger, collaborative initiative seeking to address antibody reproducibility issues by characterizing commercially available antibodies for human proteins and publishing the results openly as a resource for the scientific community. While use of antibodies and protocols vary between laboratories, we encourage readers to use this report as a guide to select the most appropriate antibodies for their specific needs.

## Introduction

Stimulator of interferon genes protein (STING1) is a conserved transmembrane protein involved in innate immune response mechanisms. ^1^^,^^2^ Activated in response to bacterial cyclic dinucleotides, it initiates a cascade of signalling events inducing the production of type I interferons and inflammatory cytokines, essential molecules to activate immune response signalling. ^1^^,^^3^^,^^4^

While STING1-mediated innate immunity is involved in defending against pathogen invasion and tumor growth, aberrant expression can disrupt immune responses, ^5^ leading to autoinflammatory diseases, cancer and neurodegenerative diseases. ^6^^–^^8^ In Parkinson’s disease models, α-synuclein aggregates enhance STING1-dependent neuroinflammation and neurodegeneration. ^9^ Due to its involvement in critical immune pathways, STING1 has become a key focus for therapeutic development.

This research is part of a broader collaborative initiative in which academics, funders and commercial antibody manufacturers are working together to address antibody reproducibility issues by characterizing commercial antibodies for human proteins using standardized protocols, and openly sharing the data. ^10^^–^^12^ Here we evaluated the performance of sixteen commercial antibodies for STING1 for use in western blot, immunoprecipitation, and immunofluorescence, enabling biochemical and cellular assessment of STING1 properties and function. The platform for antibody characterization used to carry out this study was endorsed by a committee of industry academic representatives. It consists of identifying human cell lines with adequate target protein expression and the development/contribution of equivalent knockout (KO) cell lines, followed by antibody characterization procedures using most commercially available antibodies against the corresponding protein. The standardized consensus antibody characterization protocols are openly available on Protocol Exchange, a preprint server (DOI: 10.21203/rs.3.pex-2607/v1). ^13^

The authors do not engage in result analysis or offer explicit antibody recommendations. Our primary aim is to deliver top-tier data to the scientific community, grounded in Open Science principles. This empowers experts to interpret the characterization data independently, enabling them to make informed choices regarding the most suitable antibodies for their specific experimental needs. Guidelines on how to interpret antibody characterization data found in this study are featured on the YCharOS gateway. ^14^

## Results and discussion

Our standard protocol involves comparing readouts from WT (wild type) and KO cells. ^15^^,^^16^ The first step was to identify a cell line(s) that expresses sufficient levels of a given protein to generate a measurable signal using antibodies. To this end, we examined the DepMap (Cancer Dependency Map Portal, RRID:SCR_017655) transcriptomics database to identify all cell lines that express the target at levels greater than 2.5 log _2_ (transcripts per million “TPM” + 1), which we have found to be a suitable cut-off. ^10^ The THP-1 cell line expresses the STING1 transcript at 4.1 log _2_ (TPM+1), which is above the average range of cancer cells analyzed. A *STING1* KO in THP-1 was obtained from Abcam ( Table 1). THP-1 cells are small, round human leukemia monocytic cell line commonly used to study proteins involved in immune responses. Phorbol 12-myristate-13-acetate (PMA) treatment is required to differentiate THP-1 in suspension into macrophage-like adherent cells. ^17^

**Table 1.** Summary of the cell lines used.

Institution	Catalog number	RRID (Cellosaurus)	Cell line	Genotype
Abcam	ab271147	CVCL_0006	THP-1	WT
Abcam	ab270493	CVCL_B1AK	THP-1	*STING1* KO

**Figure 1.**
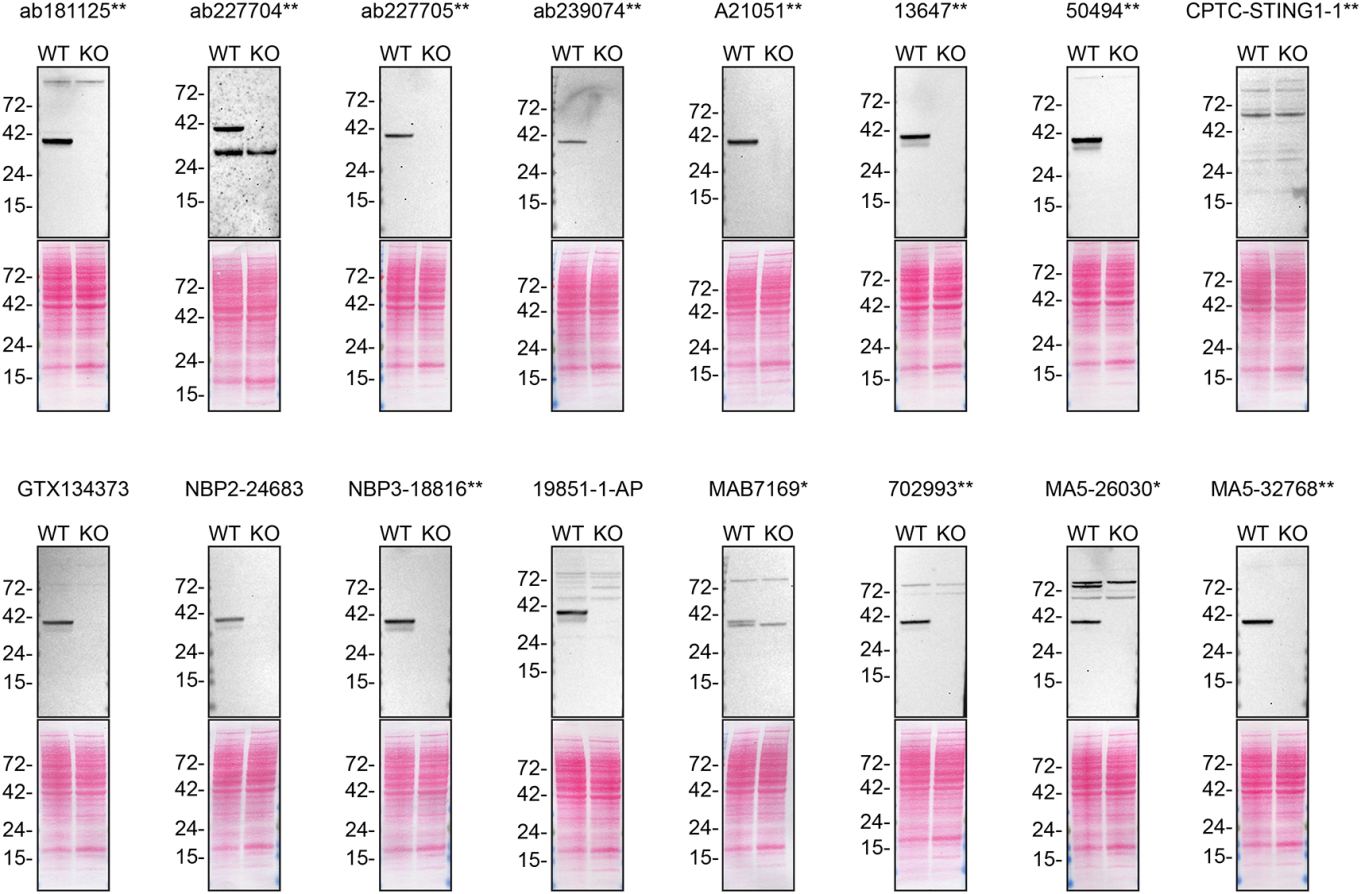
STING1 antibody screening by western blot. Lysates from PMA-treated THP-1 WT and *STING1* KO were prepared, and 40 μg of protein were processed for western blot with the indicated STING1 antibodies. The Ponceau stained transfers of each blot are presented to show equal loading of WT and KO lysates and protein transfer efficiency from polyacrylamide gels to the nitrocellulose membrane. Antibody dilutions were chosen according to the recommendations of the antibody supplier. Antibody dilution used: ab181125** at 1/1000, ab227704** at 1/200, ab227705** at 1/1000, ab239074** at 1/1000, A21051** at 1/1000, 13647** at 1/1000, 50494** at 1/1000, CPTC-STING1-1** at 1/10, GTX134373 at 1/1000, NBP2-24683 at 1/1000, NBP3-18816** at 1/1000, 19851-1-AP at 1/1000, MAB7169* at 1/1000, 702993** at 1/1000, MA5-26030* at 1/1000, MA5-32768** at 1/1000. Predicted band size: 42 kDa. *Monoclonal antibody, **Recombinant antibody.

For western blot experiments, WT and *STING1* KO protein lysates were first separated on SDS-PAGE, transferred onto nitrocellulose membranes, and then probed with sixteen STING1 antibodies in parallel ( Table 2, Figure 1).

**Table 2.** Summary of the STING1 antibodies tested.

Company	Catalog number	Lot number	RRID (Antibody Registry)	Clonality	Clone ID	Host	Concentration (μg/μl)	Vendors recommended applications
Abcam	ab181125 **	1000056-10	AB_2916053	recombinant-mono	EPR13130	rabbit	0.30	Wb, IF
Abcam	ab227704 **	1004954-2	AB_3083474	recombinant-mono	SP338	rabbit	1.50	Wb, IF
Abcam	ab227705 **	1062328-1	AB_3076486	recombinant-mono	SP339	rabbit	0.31	Wb
Abcam	ab239074 **	1001652-16	AB_3068528	recombinant-mono	EPR13130-55	rabbit	0.53	Wb, IP, IF
ABclonal	A21051 **	6100001330	AB_3083450	recombinant-mono	ARC57967	rabbit	0.29	Wb
Cell Signaling Technology	13647 **	9	AB_2732796	recombinant-mono	D2P2F	rabbit	0.06	Wb, IP
Cell Signaling Technology	50494 **	8	AB_2799375	recombinant-mono	D1V5L	rabbit	0.56	Wb, IP
DSHB	CPTC-STING1-1 **	4/27/23-15ug/mL	AB_3068346	recombinant-mono	CPTC-STING1-1	rabbit	0.02	Wb, IF
GeneTex	GTX134373	43628	AB_2887262	polyclonal	-	rabbit	0.42	Wb, IF
Novus Biologicals (Bio-Techne)	NBP2-24683	G-4	AB_2868483	polyclonal	-	rabbit	1.00	Wb, IF
Novus Biologicals (Bio-Techne)	NBP3-18816 **	COMU01	AB_3068548	recombinant-mono	2922D	rabbit	1.00	Wb, IF
Proteintech	19851-1-AP	00118975	AB_10665370	polyclonal	-	rabbit	0.70	Wb, IP, IF
R&D Systems (Bio-Techne)	MAB7169 *	CFWR0420041	AB_10971940	monoclonal	723505	mouse	0.20	Wb, IF
Thermo Fisher Scientific	702993 **	2842286	AB_2762404	recombinant-mono	2H1L5	rabbit	0.50	Wb
Thermo Fisher Scientific	MA5-26030 *	YJ4087701	AB_2725437	monoclonal	OTI4H1	mouse	1.00	Wb, IF
Thermo Fisher Scientific	MA5-32768 **	YJ4089249A	AB_2810045	recombinant-mono	JM03-47	rabbit	1.00	Wb

Wb = western blot; IF = immunofluorescence; IP = immunoprecipitation, DSHB = Developmental Studies Hybridoma Bank.

* Monoclonal antibody.

** Recombinant antibody.

**Figure 2.**
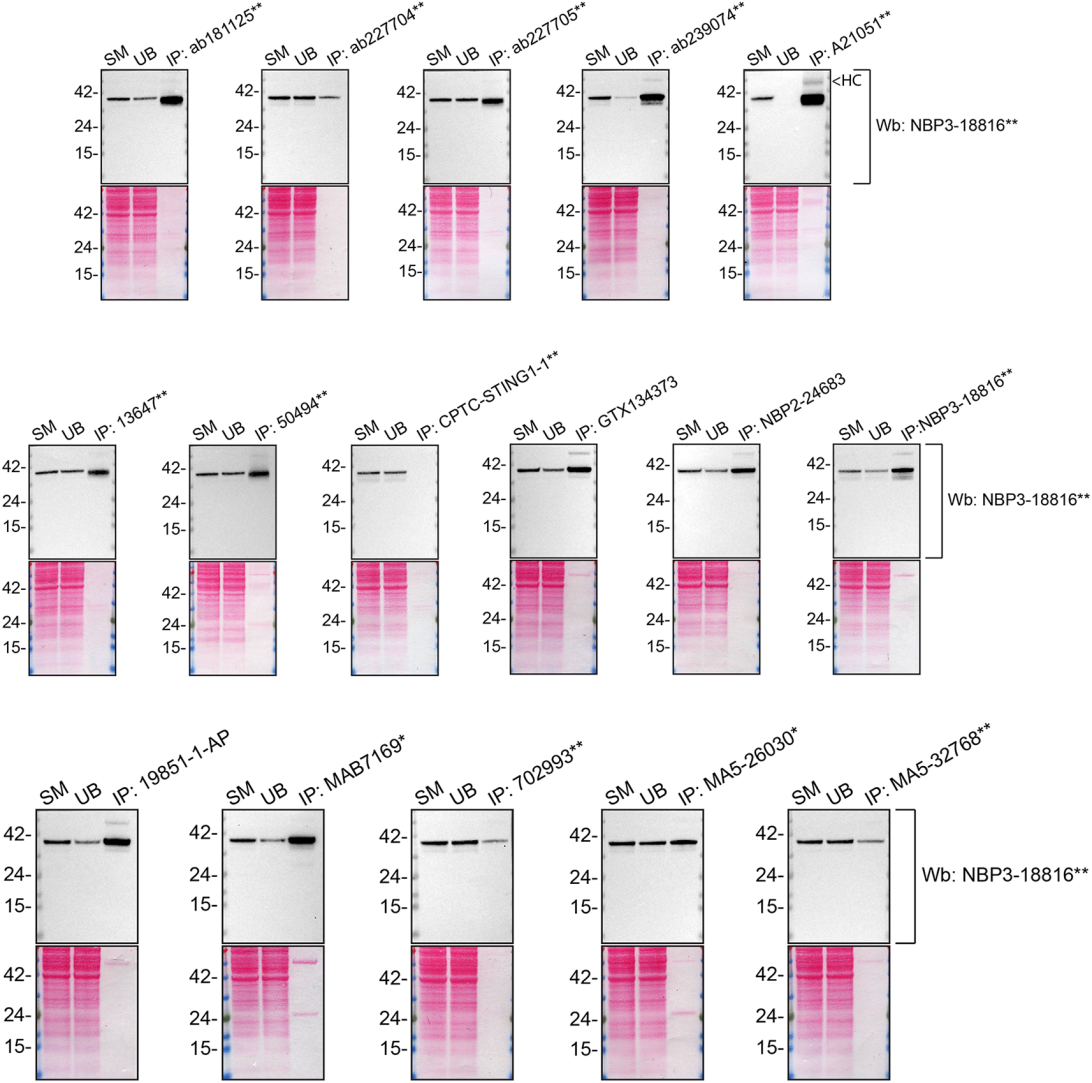
STING1 antibody screening by immunoprecipitation. Lysates from PMA-treated THP-1 WT were prepared, and immunoprecipitation was performed using 1.0 mg of lysate and 2.0 μg of the indicated STING1 antibodies pre-coupled to Dynabeads protein A or protein G. Samples were washed and processed for western blot with the indicated STING1 antibody. For western blot, NBP3-18816** was used at 1/1000. The Ponceau stained transfers of each blot are shown. SM = 4% starting material; UB = 4% unbound fraction; IP = immunoprecipitate, HC = antibody heavy chain. *Monoclonal antibody, **Recombinant antibody.

We then assessed the capability of all sixteen antibodies to capture STING1 from THP-1 protein extracts using immunoprecipitation techniques, followed by western blot analysis. For the immunoblot step, a specific STING1 antibody identified previously (refer to Figure 1) was selected. Equal amounts of the starting material (SM) and unbound fractions (UB), as well as the whole immunoprecipitate (IP) eluates were separated by SDS-PAGE ( Figure 2).

**Figure 3.**
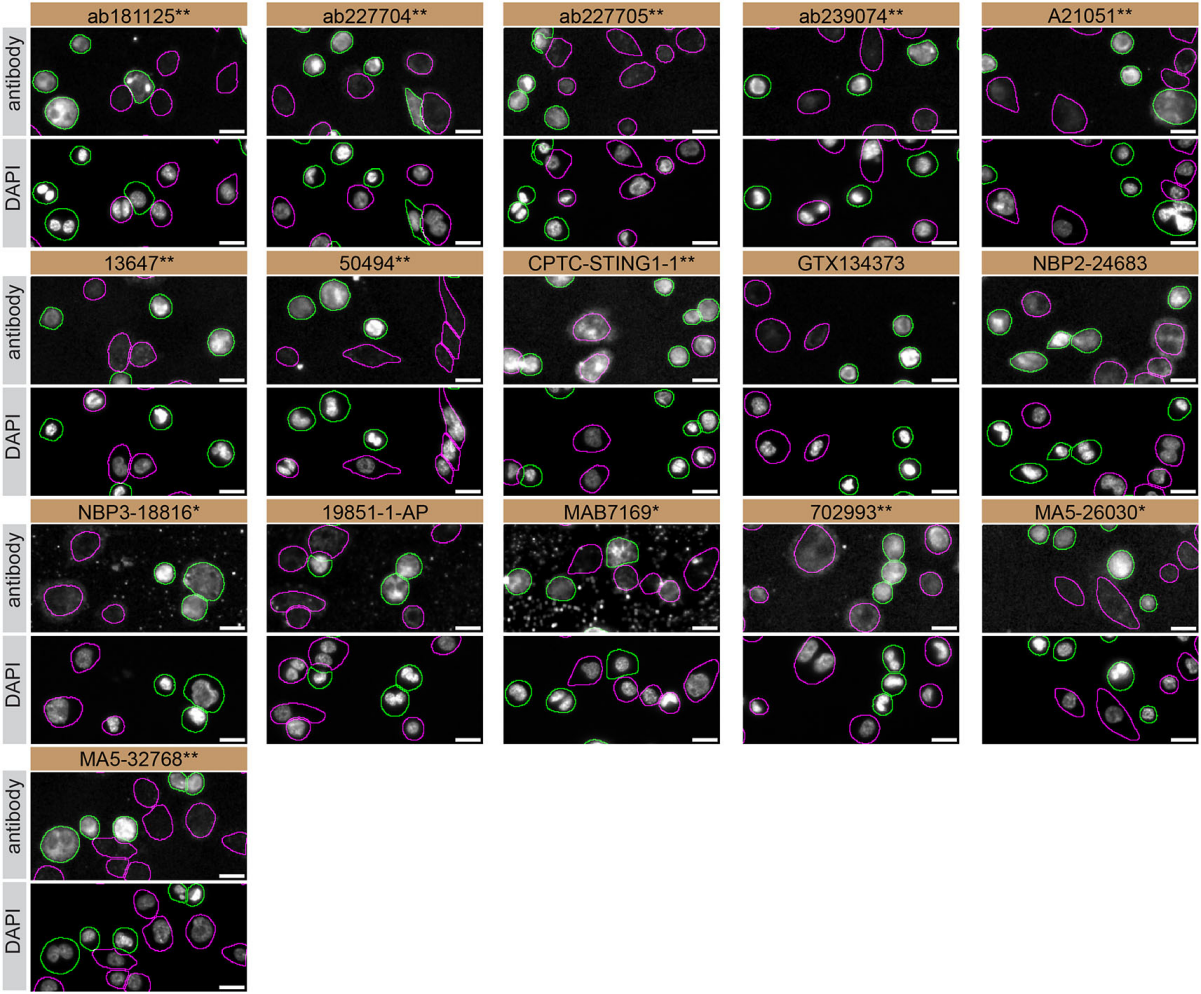
STING1 antibody screening by immunofluorescence. PMA-treated THP-1 WT and *STING1* KO cells were labelled with a green or a far-red fluorescent dye, respectively. WT and KO cells were mixed and plated to a 1:1 ratio in a 96-well plate with optically clear flat-bottom. Cells were stained with the indicated STING1 antibodies and with the corresponding Alexa-fluor 555 coupled secondary antibody including DAPI. Acquisition of the blue (nucleus-DAPI), green (identification of WT cells), red (antibody staining) and far-red (identification of KO cells) channels was performed. Representative images of the merged blue and red (grayscale) channels are shown. WT and KO cells are outlined with green and magenta dashed line, respectively. When an antibody was recommended for immunofluorescence by the supplier, we tested it at the recommended dilution. The rest of the antibodies were tested at 1 and 2 μg/ml and the final concentration was selected based on the detection range of the microscope used and a quantitative analysis not shown here. Antibody dilution used: ab181125** at 1/300, ab227704** at 1/1000, ab227705** at 1/300, ab239074** at 1/500, A21051** at 1/300, 13647** at 1/60, 50494** at 1/500, CPTC-STING1-1** at 1/20, GTX134373 at 1/1000, NBP2-24683 at 1/1000, NBP3-18816** at 1/1000, 19851-1-AP at 1/200, MAB7169* at 1/200, 702993** at 1/250, MA5-26030* at 1/1000, MA5-32768** at 1/1000. Bars = 10 μm. *Monoclonal antibody, **Recombinant antibody.

For immunofluorescence, sixteen antibodies were screened using a mosaic strategy. First, THP-1 WT and *STING1* KO cells were labelled with different fluorescent dyes in order to distinguish the two cell lines, and the STING1 antibodies were evaluated. Both WT and KO lines imaged in the same field of view to reduce staining, imaging and image analysis bias ( Figure 3). Quantification of immunofluorescence intensity in hundreds of WT and KO cells was performed for each antibody tested, and the images presented in Figure 3 are representative of this analysis. ^13^

In conclusion, we have screened sixteen STING1 commercial antibodies by western blot, immunoprecipitation, and immunofluorescence by comparing the signal produced by the antibodies in human THP-1 WT and *STING1* KO cells. To assist users in interpreting antibody performance, Table 3 outlines various scenarios in which antibodies may perform in all three applications. Several high-quality and renewable antibodies that successfully detect STING1 were identified in all applications. Researchers who wish to study STING1 in a different species are encouraged to select high-quality antibodies, based on the results of this study, and investigate the predicted species reactivity of the manufacturer before extending their research.

**Table 3.** Illustrations to assess antibody performance in western blot, immunoprecipitation and immunofluorescence.

Western blot	Immunoprecipitation	Immunofluorescence
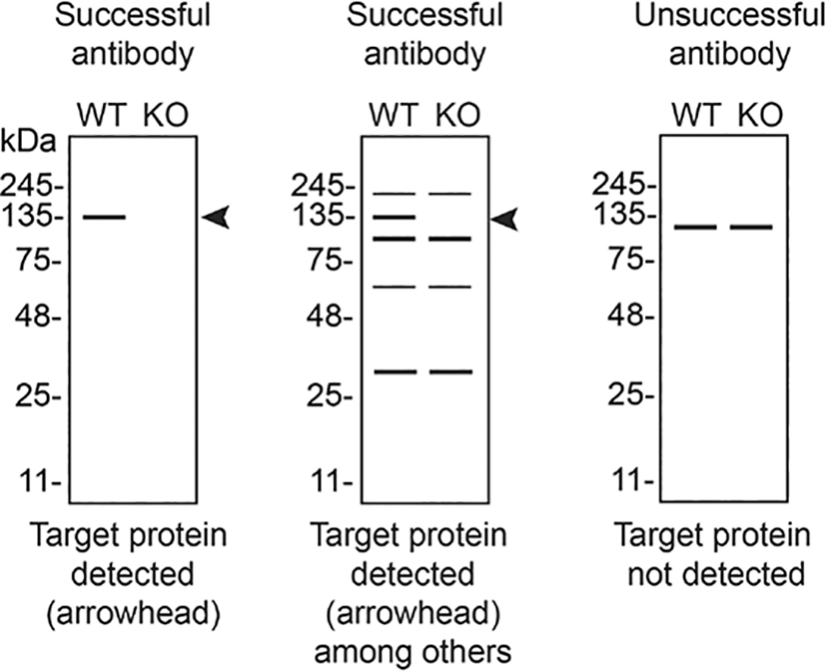	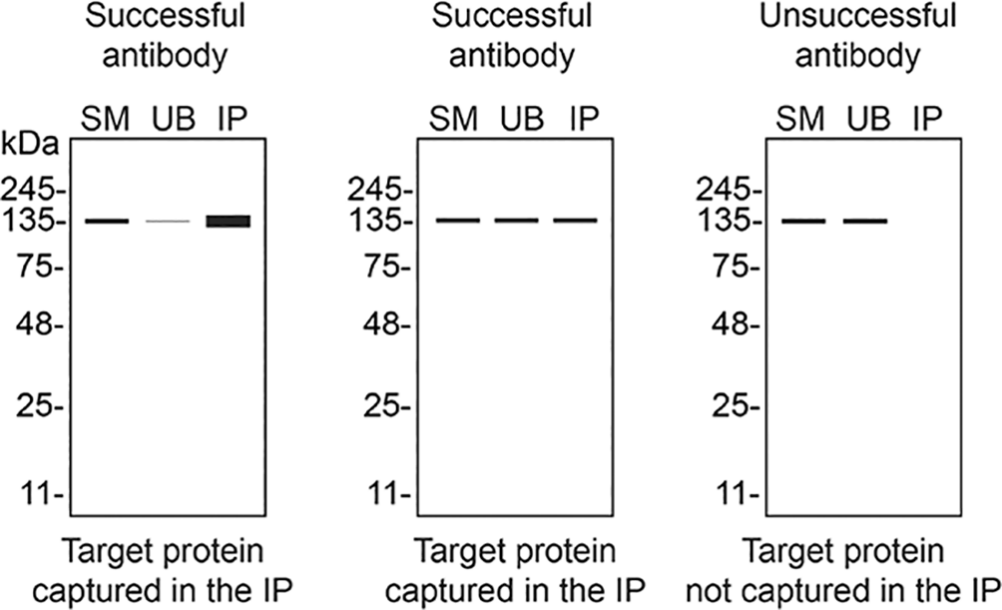	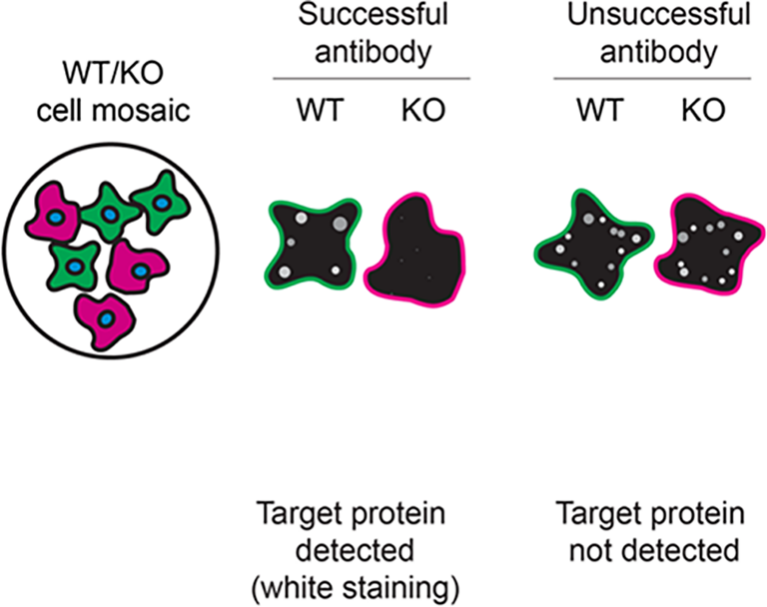

This table was reproduced with permission from Ayoubi et al., Elife, 2023. ^10^

The underlying data for this study can be found on Zenodo, an open-access repository for which YCharOS has its own collection of antibody characterization reports. ^18^^,^^19^

### Limitations

Inherent limitations are associated with the antibody characterization platform used in this study. Firstly, the YCharOS project focuses on renewable (recombinant and monoclonal) antibodies and does not test all commercially available STING1 antibodies. YCharOS partners provide approximately 80% of all renewable antibodies, but some top-cited polyclonal antibodies may not be available through these partners.

Secondly, the YCharOS effort employs a non-biased approach that is agnostic to the protein for which antibodies have been characterized. The aim is to provide objective data on antibody performance without preconceived notions about how antibodies should perform or the molecular weight that should be observed in western blot. As the authors are not experts in STING1, only a brief overview of the protein’s function and its relevance in disease is provided. STING1 experts are responsible for analyzing and interpreting observed banding patterns in western blots and subcellular localization in immunofluorescence.

Thirdly, YCharOS experiments are not performed in replicates primarily due to the use of multiple antibodies targeting various epitopes. Once a specific antibody is identified, it validates the protein expression of the intended target in the selected cell line, confirms the lack of protein expression in the KO cell line and supports conclusions regarding the specificity of the other antibodies. All experiments are performed using master mixes, and meticulous attention is paid to sample preparation and experimental execution. In immunofluorescence, the use of two different concentrations serves to evaluate antibody specificity and can aid in assessing assay reliability. In instances where antibodies yield no signal, a repeat experiment is conducted following titration. Additionally, our independent data is performed subsequently to the antibody manufacturers internal validation process, therefore making our characterization process a repeat.

Lastly, as comprehensive and standardized procedures are respected, any conclusions remain confined to the experimental conditions and cell line used for this study. The use of a single cell type for evaluating antibody performance poses as a limitation, as factors such as target protein abundance significantly impact results. ^13^ Additionally, the use of cancer cell lines containing gene mutations poses a potential challenge, as these mutations may be within the epitope coding sequence or other regions of the gene responsible for the intended target. Such alterations can impact the binding affinity of antibodies. This represents an inherent limitation of any approach that employs cancer cell lines.

## Methods

The standardized protocols used to carry out this KO cell line-based antibody characterization platform was established and approved by a collaborative group of academics, industry researchers and antibody manufacturers. The detailed materials and step-by-step protocols used to characterize antibodies in western blot, immunoprecipitation and immunofluorescence are openly available on Protocol Exchange, a preprint server (DOI: 10.21203/rs.3.pex-2607/v1). ^13^ Brief descriptions of the experimental setup used to carry out this study can be found below.

### Cell lines and antibodies used

Cell lines used and primary antibodies tested in this study are listed in Tables 1 and 2, respectively. To ensure that the cell lines and antibodies are cited properly and can be easily identified, we have included their corresponding Research Resource Identifiers, or RRID. ^20^^,^^21^ Cells were cultured in RPMI 1640 (Gibco, cat. number A1049101) containing 10% fetal bovine serum (Wisent, cat. number 080450), 0.05 mM 2-Mercaptoethanol (Gibco, cat. number 21985023, 2 mM L-glutamine (Wisent cat. number 609-065), 100 IU penicillin and 100 μg/ml streptomycin (Wisent cat. number 450201). THP-1 WT and *STING1* KO cells were treated with 200 ng/ml PMA (Abcam, cat. number ab147465) for 2 days. 200 ng/ml of PMA was added to fresh medium in both day 1 and day 2. ^17^ Peroxidase-conjugated goat anti-rabbit and anti-mouse antibodies (Thermo Fisher Scientific, cat. number 65-6120 and 62-6520) and Peroxidase-conjugated Protein A (MilliporeSigma, cat. number P8651) were used in western blot and immunoprecipitation. Alexa-555-conjugated goat anti-rabbit and anti-mouse secondary antibodies (Thermo Fisher Scientific, cat. number A-21429 and A-21424) were used in immunofluorescence.

### Antibody screening by western blot

PMA-treated THP-1 WT and *STING1* KO cells (listed in Table 1) were collected in RIPA buffer (25mM Tris-HCl pH 7.6, 150mM NaCl, 1% NP-40, 1% sodium deoxycholate, 0.1% SDS) (Thermo Fisher Scientific, cat. number 89901) supplemented with 1x protease inhibitor cocktail mix (MilliporeSigma, cat. number P8340). Lysates were sonicated briefly and incubated for 30 min on ice. Lysates were spun at ~110,000 × *g* for 15 min at 4°C and equal protein aliquots of the supernatants were analyzed by SDS-PAGE and western blot. BLUelf prestained protein ladder (GeneDireX, cat. number PM008-0500) was used.

Western blots were performed with precast midi 10% Bis-Tris polyacrylamide gels (Thermo Fisher Scientific, cat. number WG1201BOX) ran with MES SDS buffer (Thermo Fisher Scientific, cat. number NP000202), loaded in LDS sample buffer (Thermo Fisher Scientific, cat. number NP0008) with 1× sample reducing agent (Thermo Fisher Scientific, cat. number NP0009) and transferred on nitrocellulose membranes. Proteins on the blots were visualized with Ponceau S staining (Thermo Fisher Scientific, cat. number BP103-10) which is scanned to show together with individual western blot. Blots were blocked with 5% milk for 1 hr, and antibodies were incubated overnight at 4°C with 5% milk in TBS with 0.1% Tween 20 (TBST) (Cell Signaling Technology, cat. number 9997). Following three washes with TBST, the peroxidase conjugated secondary antibody was incubated at a dilution of ~0.2 μg/ml in TBST with 5% milk for 1 hr at room temperature followed by three washes with TBST. Membranes were incubated with Pierce ECL (Thermo Fisher Scientific, cat. number 32106) or Clarity Western ECL Substrate (Bio-Rad, cat. number 1705061) prior to detection with the iBright™ CL1500 Imaging System (Thermo Fisher Scientific, cat. number A44240).

### Antibody screening by immunoprecipitation

Antibody-beads conjugates were prepared by adding 2 μg of antibody (with an exception 20 μl of antibody CPTC-STING1-1** and 20 μl of antibody 13647**) to 500 μl of Pierce IP Lysis Buffer (Thermo Fisher Scientific, cat. number 87788) in a microcentrifuge tube, together with 30μl of Dynabeads protein A- (for rabbit antibodies) or protein G- (for mouse antibodies) (Thermo Fisher Scientific, cat. number 10002D and 10004D, respectively). Tubes were rocked for ~1 hr at 4°C followed by two washes to remove unbound antibodies.

PMA-treated THP-1 WT cells were collected in Pierce IP buffer (25 mM Tris-HCl pH 7.4, 150 mM NaCl, 1 mM EDTA, 1% NP-40 and 5% glycerol) supplemented with protease inhibitors. Lysates were rocked for 30 min at 4°C and spun at 110,000 × *g* for 15 min at 4°C. 0.5 ml aliquots at 2.0 mg/ml of lysate were incubated with an antibody-bead conjugate for ~1 hr at 4°C. The unbound fractions were collected, and beads were subsequently washed three times with 1.0 ml of IP lysis buffer and processed for SDS-PAGE and western blot on precast midi 10% Bis-Tris polyacrylamide gels. Protein A:HRP (MilliporeSigma, cat. number P8651) was used as a secondary detection system at a concentration of 2.0 μg/ml.

### Antibody screening by immunofluorescence

PMA-treated THP-1 WT and *STING1* KO cells were labelled with a green and a far-red fluorescence dye, respectively (Thermo Fisher Scientific, cat. number C2925 and C34565, respectively). The nuclei were labelled with DAPI (Thermo Fisher Scientific, cat. Number D3571) fluorescent stain. WT and KO cells were plated in a 96-well plate with optically clear flat-bottom. (Perkin Elmer, cat. number 6055300) as a mosaic and incubated for 24 hrs in a cell culture incubator, 37 ^o^C, 5% CO _2_. Cells were fixed in 4% paraformaldehyde (PFA) (Beantown chemical, cat. number 140770-10 ml) in phosphate buffered saline (PBS) (Wisent, cat. number 311-010-CL) for 15 min at room temperature and then washed 3 times with PBS. Cells were permeabilized in PBS with 0.1% Triton X-100 (Thermo Fisher Scientific, cat. number BP151-500) for 10 min at room temperature and blocked with PBS with 5% BSA, 5% goat serum (Gibco, cat. number 16210-064) and 0.01% Triton X-100 for 30 min at room temperature. Cells were incubated with IF buffer (PBS, 5% BSA, 0.01% Triton X-100) containing the primary STING1 antibodies overnight at 4°C. Cells were then washed 3 × 10 min with IF buffer and incubated with corresponding Alexa Fluor 555-conjugated secondary antibodies in IF buffer at a dilution of 1.0 μg/ml for 1 hr at room temperature with DAPI. Cells were washed 3 × 10 min with IF buffer and once with PBS.

Images were acquired on an ImageXpress micro confocal high-content microscopy system (Molecular Devices), using a 20× NA 0.95 water immersion objective and scientific CMOS cameras, equipped with 395, 475, 555 and 635 nm solid state LED lights (lumencor Aura III light engine) and bandpass filters to excite DAPI, Cellmask Green, Alexa-555 and Cellmask Red, respectively. Images had pixel sizes of 0.68 × 0.68 microns, and a z-interval of 4 microns. For analysis and visualization, shading correction (shade only) was carried out for all images. Then, maximum intensity projections were generated using 3 z-slices. Segmentation was carried out separately on maximum intensity projections of Cellmask channels using CellPose 1.0, and masks were used to generate outlines and for intensity quantification. ^22^ Figures were assembled with Adobe Illustrator.

## Data Availability

Zenodo: Antibody Characterization Report for STING1, https://doi.org/10.5281/zenodo.11582350. ^18^ Zenodo: Dataset for the stimulator of interferon genes protein (STING1) antibody screening study, https://doi.org/10.5281/zenodo.12761294. ^19^ Data are available under the terms of the Creative Commons Attribution 4.0 International license (CC-BY 4.0).
