# Supplementary material for: A guide to selecting high-performing antibodies for STING1 (Uniprot ID: Q86WV6) for use in western blot, immunoprecipitation, and immunofluorescence
Source: F1000Res. 2025 Jan 2;13:1049. Originally published 2024 Sep 12. [Version 2] doi: 10.12688/f1000research.155929.2 (PMC12175898; doi:10.12688/f1000research.155929.2)
Supplement: Peer review history

# Reviewer report: Reviewer response for version 2

**DOI:** 10.5256/f1000research.175918.r356517

**Published:** 2025-02-05

**Authors:** John LaCava, Mathias Nielsen

**Affiliations:** 1 Laboratory of Cellular and Structural Biology, The Rockefeller University (Ringgold ID: 5929), New York, New York, USA; 2 University Medical Centre Groningen, The Rockefeller University, Groningen, The Netherlands

The research presented by Moleón et al. is directed at demonstrating examples of standard operating procedures for the rigorous selection of antibodies to use in western blotting, immunoprecipitation, and immunofluorescence applications. The guidance given is sound and pertains to the principled selection of antibodies for these applications based on well-articulated criteria: the presence of signal in the WT and absence of signal in the cognate KO cell line (specificity in western); the depletion of signal in the unbound compared to the starting material along with the appearance of signal in the IP eluate (affinity to the target in IP), the presence of signal in the WT and absence of signal in the KO (specificity in IF). The authors are careful to state that the quality and pattern of the results are not necessarily transferable to other cell lines and experimental conditions, and are therefore representative of what one should expect from these reagents, given the experimental framework used.

 There are some intermediate or marginal cases presented within some of the data and various ways to interpret those. Some extended discussion about the meaning of such cases would be welcomed, but is not necessary to justify the work. Effectively, this article could help investigators looking for promising STING1 antibodies to short list pre-validated candidates, taken from this list, for screening in their applications, under their own experimental conditions - and evaluate those results in context, with the support of the comparative context provided here.

 It would have been satisfying to have mass spectrometry included in the analysis of the IP results - including the IP done in the WT vs KO for selected antibodies - or, at least to discuss a role for mass spectrometry in antibody validation for IP / co-IP (given that this is such a common application), but this appears to be outside of the intended scope of the research and not necessary to justify the work. 

 This work, and work like it, contributes to more reproducible research by instructing investigators on principled ways  to evaluate antibodies and, in this specific case, providing them with data that reduces their burden of evaluation for commercially available anti-STING1 products. 

 I believe the following critiques apply and should be addressed:

 1. Failure to indicate the units of the ingredients listed by %. Some will be e.g., by volume, such as 1% (v/v) NP-40, whereas others will be e.g., by weight, such as 0.1% (w/v) SDS - unlabeled % units appear through the methods for multiple reagents and it is best to ensure the reader is provided this info to complete the methods. 

 2. It seems that the experiments (WB, IP, and IF) are performed on PMA-treated (differentiated) THP-1 cells. The authors state that the PMA-treatment is required for differentiation into macrophage-like cells, but it is never explained why this treatment is relevant to the different experiments. The authors had already screened cell lines specifically for high STING1 expression and had chosen the THP-1 cell line - so why is the PMA-treatment needed? Is the expression of STING1 higher or lower in the differentiated cell? For IF experiments the treatment could be necessary, as the THP-1 cell line is a suspension cell that becomes adherent after the PMA treatment. So is the point to use the same input material / cell type for all experiments? One sentence could help clear this up.

 3. In the Methods section under 'Antibody screening by western blot' the authors write: "Membranes were incubated with Pierce ECL (Thermo Fisher Scientific, cat. number 32106) or Clarity Western ECL Substrate (Bio-Rad, cat. number 1705061) prior to detection with the iBright™ CL1500 Imaging System (Thermo Fisher Scientific, cat. number A44240)." Why did the authors use two different ECL reagents? And does this affect the interpretation of the WB results? Since the results are presented as individual stand along panels - this should have no effect - but in the event that different panels are being compared, this would be a potential issues. The fact that two different reagents are used is not a problem, but it would be nice to add one line to the method or discussion stating that the blots are not intended to be compared to one another, per se, except through ratiometric means (e.g. UB/SM or IP/SM) and only qualitatively or semi-quantitatively (they mention these are only single replicate experiments, so, they would not be intended to be used quantitatively anyway). In essence, some verbiage on how these results can be used between panels (if at all), not just within panels, would be desirable.

Are sufficient details of methods and materials provided to allow replication by others?

Partly

Is the rationale for creating the dataset(s) clearly described?

Yes

Are the datasets clearly presented in a useable and accessible format?

Yes

Are the protocols appropriate and is the work technically sound?

Yes

Reviewer Expertise:

Protein and nucleic acids biochemistry, proteomics / interactomics.

We confirm that we have read this submission and believe that we have an appropriate level of expertise to confirm that it is of an acceptable scientific standard, however we have significant reservations, as outlined above.

# Reviewer report: Reviewer response for version 2

**DOI:** 10.5256/f1000research.175918.r356518

**Published:** 2025-01-23

**Authors:** Sheng-ce Tao

**Affiliations:** 1 Shanghai Jiao Tong University, Shanghai, China

Because of the importance of STING, high quality antibody is a needed. This study compared sixteen commercially available STING1 antibodies for use in western blot, immunoprecipitation, and immunofluorescence. The antibodies were tested using standardized protocols in knockout cell lines and isogenic parental controls to assess their reliability. The aim of this study is helping researchers select the most suitable antibodies for perform STING related studies. Overall, I endorse the acceptance of this study.

 1. What are the criteria for selecting the 16 commercial antibodies? Please clarify.

 2. This study only tested one cell line (THP-1). It is not necessary that the conclusion from one cell generally also works among other cell lines. 

 3. Since one of the major application of these validated antibodies is WB, it is highly possible that these antibodies recognize linear epitopes on STING. In this case, mapping the epitopes (for example, using AbMap technology), and then judge the specificity by epitope searching across the whole proteome will be a more general  approach.

Are sufficient details of methods and materials provided to allow replication by others?

Yes

Is the rationale for creating the dataset(s) clearly described?

Yes

Are the datasets clearly presented in a useable and accessible format?

Yes

Are the protocols appropriate and is the work technically sound?

Yes

Reviewer Expertise:

Antibody technology, proteomics

I confirm that I have read this submission and believe that I have an appropriate level of expertise to confirm that it is of an acceptable scientific standard.

# Reviewer report: Reviewer response for version 2

**DOI:** 10.5256/f1000research.175918.r355436

**Published:** 2025-01-13

**Authors:** Jieya Shao

**Affiliations:** 1 Washington University in St Louis, St. Louis, Missouri, USA

I approve this revised report.

Are sufficient details of methods and materials provided to allow replication by others?

Yes

Is the rationale for creating the dataset(s) clearly described?

Yes

Are the datasets clearly presented in a useable and accessible format?

Yes

Are the protocols appropriate and is the work technically sound?

Yes

Reviewer Expertise:

molecular and cellular biology

I confirm that I have read this submission and believe that I have an appropriate level of expertise to confirm that it is of an acceptable scientific standard.

# Reviewer report: Reviewer response for version 1

**DOI:** 10.5256/f1000research.171164.r333636

**Published:** 2024-11-08

**Authors:** Jieya Shao

**Affiliations:** 1 Washington University in St Louis, St. Louis, Missouri, USA

In this article the authors systematically compared the performance and specificity of sixteen commercial anti-STING1 antibodies using three different experimental approaches (Western blot, immunoprecipitation, and immunofluorescence staining). This study is part of a large collaborative initiative to characterize commercial antibodies for different human proteins and offer unbiased guidance to researchers who can select the best antibodies for their specific experimental objectives based on their own interpretations. As a Data Note, this paper successfully achieved its goal with rigorously collected high-quality data, and the results are self-explanatory and useful for the research community.

 However, there are a few minor points to address. First, the authors wrote in the Introduction section that “activated in response to bacterial cyclic dinucleotides, STING1 initiates a cascade of signaling events”. This statement seems incorrect as cGAMP can be generated endogenously by cGAS rather than derived from bacteria. Second, the statement that aberrant expression of STING1 can lead to cancer needs clarification. Third, in Fig.2, the bottom panel of images are larger than the rest. Fourth, the images in Fig.3 can be rearranged (e.g. 4 x 4) for better presentation.

Are sufficient details of methods and materials provided to allow replication by others?

Yes

Is the rationale for creating the dataset(s) clearly described?

Yes

Are the datasets clearly presented in a useable and accessible format?

Yes

Are the protocols appropriate and is the work technically sound?

Yes

Reviewer Expertise:

molecular and cellular biology

I confirm that I have read this submission and believe that I have an appropriate level of expertise to confirm that it is of an acceptable scientific standard, however I have significant reservations, as outlined above.

# Response

**Published:** 2024-12-12

**Authors:** Carl Laflamme

**Affiliations:** McGill University, Canada

Dear Dr. Shao,

 Thank you for taking the time to review our STING1 antibody characterization report. We greatly appreciate your feedback.

 This report is part of a global initiative aimed at characterizing antibodies for every human protein. While the authors are experts in antibody development, we acknowledge that our expertise does not extend specifically to the STING1 protein. In light of your comments, we have revised the introduction to ensure greater accuracy and clarity regarding STING1.

 Additionally, we have modified Figures 2 and 3 in accordance with your recommendations. We trust these changes address your concerns and enhance the quality of our report.

 Thank you once again for your thoughtful insights.

 Best regards,

 Carl

# Reviewer report: Reviewer response for version 1

**DOI:** 10.5256/f1000research.171164.r333635

**Published:** 2024-11-07

**Authors:** Karen Bowman

**Affiliations:** 1 University of Leicester, Leicester, England, UK

The study involved characterisation of sixteen commercial antibodies, from nine different suppliers, for the stimulator of interferon genes protein (STING1) for use in western blot, immunoprecipitation, and immunofluorescence. The study formed part of the wider YCharOS collaboration to characterise commercial antibodies for human proteins using standardized protocols with the intention of improving antibody reproducibility issues. As a Data Note, it allows the direct comparison of the performance of commercially available antibodies to STING1 protein to aid scientists in choosing the most appropriate antibody for their studies. This would be of use in several fields such as study of innate immunity, autoinflammatory diseases, cancer and neurodegenerative diseases.

 A committee of industry and academic representatives have endorsed the platform used, and the protocols used appear to be consistent with those in general use. The platform consisted of identification of a human cell line suitable for antibody characterisation studies i.e., with adequate levels of the STING1 protein to generate a measurable signal. They found that the commercially available THP-1 WT cell line expresses the STING1 transcript at a level above the average range of cancer cells analysed, and therefore, it was a logical choice for their study. The matched isogenic knockout control cell line, THP-1 *STING1* KO was used as an appropriate negative control. The final step was a series of antibody characterisation procedures, limited to the most common research uses of antibodies. The interpretation of the results is left to the reader, with the study providing unbiased guidance. Limitations of the study are clearly stated. The summary Table 3 with illustrations to assess antibody performance in western blot, immunoprecipitation and immunofluorescence, is very helpful.

 A minor point to note. An ‘at a glance’ summary table encompassing the outcomes for each antibody would be useful.

 In conclusion, the study will be a useful resource when choosing the most appropriate antibody for study of the STING1 protein.

Are sufficient details of methods and materials provided to allow replication by others?

Yes

Is the rationale for creating the dataset(s) clearly described?

Yes

Are the datasets clearly presented in a useable and accessible format?

Yes

Are the protocols appropriate and is the work technically sound?

Yes

Reviewer Expertise:

Cell biology. Drug discovery and development.

I confirm that I have read this submission and believe that I have an appropriate level of expertise to confirm that it is of an acceptable scientific standard.
